# Comment on Di Marco, N., Kaufman, J., Rodda, C.P. Shedding Light on Vitamin D Status and Its Complexities during Pregnancy, Infancy and Childhood: An Australian Perspective. *Int. J. Environ. Res. Public Health* 2019, *16* (4), 538, doi:10.3390/ijerph16040538

**DOI:** 10.3390/ijerph16081373

**Published:** 2019-04-16

**Authors:** Barbara J Boucher

**Affiliations:** Blizard Institute, Queen Mary University of London, London E12AT, UK; bboucher@doctors.org.uk

In their report on the roles of vitamin D status during early life development in Australia, Di Marco et al. suggest that the use of cod liver oil can no longer be recommended due to its high retinol content (quoted at 4080 micrograms (12,240 IU)/tablespoonful), according to data from the USA (accessed 9 February 2019) [1]; however, most countries sell preparations that have been purified and deodorized, with removal of both toxins and fat-soluble vitamins before vitamin A and D replacement at permissible levels [2] (e.g., containing retinol at 400 micrograms (1200 IU)/capsule (Boots maxi strength cod liver oil) or 800 micrograms (2400 IU)/capsule (Club VITS) in the UK). Commonly used Australian preparations provide reasonable amounts of vitamin A (e.g., 300 micrograms (1000 IU)/capsule from Blackmores, or 187.5 micrograms (~560.IU)/capsule) [3]. Cod liver oil, offered free to all pregnant women, nursing mothers, and children <5 years old during and after World War 2 in Britain, was banned from ante-natal clinics in the 1990s following recognition of retinol teratogenicity in offspring both experimentally and with maternal intakes >1200 microg (>4000 IU)/day in humans [4]. No replacement vitamin D was provided, and deficiency became common in UK pregnancies, with resurgence of neonatal and childhood rickets (mainly in dark-skinned people, solely breast-fed babies, or unsuitable infant ‘solids’) and fatalities from undiagnosed vitamin D deficiency causing neonatal hypocalcemic status epilepticus and cardiomyopathic heart failure [5]. If cod liver oil preparations with lower vitamin A contents are generally available from suppliers in Australia and in other countries, then the vitamin D they contain could contribute to the avoidance of vitamin D deficiency without risking vitamin A toxicity.
